# Longitudinal functional and neuropsychological 2-year follow-up after intensive care admission for multisystem inflammatory syndrome in children

**DOI:** 10.1007/s00431-025-06396-y

**Published:** 2025-09-15

**Authors:** Naomi Ketharanathan, Corinne M. P. Buysse, Thomas C. Seijbel, José Hordijk, Lotte Bos, Koen Joosten, Matthijs de Hoog, Karolijn Dulfer

**Affiliations:** 1 Department of Neonatal and Pediatric Intensive Care, Division of Pediatric Intensive Care, Erasmus MC Sophia Children’s Hospital, Wytemaweg 80, Rotterdam, 3015 CN The Netherlands; 2 Department of Child and Adolescent Psychiatry/Psychology, Erasmus MC Sophia Children’s Hospital, Wytemaweg 80, 3015 CN Rotterdam, The Netherlands

**Keywords:** SARS-CoV-2, Covid-19, Follow-up, Pediatric, Quality of life

## Abstract

The purpose of this study is to investigate long-term morbidity (physical, psychosocial, neurocognitive, quality of life (QoL)) in critical care patients with multisystem inflammatory syndrome in children (MIS-C). This prospective study examined MIS-C critical care admissions (May 2020–March 2022). Outcomes were determined by semi-structured interview, functional assessment scores, self- and parent-reported questionnaires, and extensive neurocognitive testing at 3 and 24 months after pediatric intensive care unit (PICU) admission. There was no control group, but general population norm data was used. Thirty-six MIS-C PICU admissions were included (median [IQR] age 10.1 [7.9–13.6] years, 72% male). Median [IQR] PICU stay was 3 days [2–4] and all survived; 81% (29/36) attended 24 months follow-up. Functional assessment scores were favorable in most. Forty-five percent reported ≥ 1 functional complaint (e.g., fatigue (23%) or exercise intolerance (17%)). Fourteen percent had not resumed full-time school, and grade adjustments were necessary in 10%. Psychosocial, neurocognitive, and QoL outcomes were comparable to the general population except for visual memory and sustained attention.

*Conclusions*: Repeated, multidimensional validated neuropsychological testing in MIS-C PICU patients was comparable to the norm population, except for visual memory and sustained attention. Functional assessment scores were mostly favorable. However, self-reported complaints remained prevalent (45%) and school participation was not possible in 14%. This discrepancy (validated vs. self-reported outcome measures) underlines the need for standardized physical testing and age- and community-matched control groups to establish an association with disease-related factors, especially during unique social circumstances. Overall, structured PICU longitudinal follow-up identifies specific domains for rehabilitation to address morbidity and enhance social participation.

**Table:** 

**What is Known:** *• MIS-C is a multi organ inflammatory response to a SARS-CoV-2 infection with varying severity ranging from subclinical to critical illness.* *• The retention of knowledge from this training is a point of concern for parents.*	
**What is New:** *• Multidimensional, longitudinal outcome of MIS-C critical care survivors showed validated functional scores and repeated neuropsychological outcomes comparable to the norm, with the exception of visual memory and sustained attention. Self-reported complaints remained prevalent (45%).* *• Longitudinal, multidisciplinary follow-up programs are crucial for (novel) disease trajectory understanding and individualized rehabilitation programs.*

**Supplementary Information:**

The online version contains supplementary material available at 10.1007/s00431-025-06396-y.

## Introduction

Shortly after the onset of the COVID-19 pandemic, reports emerged describing a surge of children with a constellation of symptoms two to six weeks after a (subclinical) SARS-CoV-2 infection [1, 2]. This disease entity has since been defined as multisystem inflammatory syndrome in children (MIS-C) by the World Health Organization (WHO) [3]. The bandwidth of clinical severity was variable and characterized by multi-organ, systemic inflammation. This necessitated pediatric intensive care unit (PICU) admission in up to 74% of hospitalized cases in the first pandemic year for supportive measures (vasoactive support in up to 80%, mechanical ventilation in up to 15%) and immunomodulatory therapies [4–7]. Mortality varied, being up to 9% in low- to middle-income countries and 1.8% in high-income countries, depending on the time point within the first pandemic year [8]. The potentially severe multi-organ inflammation in this relatively novel disease has led to concerns regarding long-term (> 1 year) morbidity. Short- to medium-term MIS-C outcome studies (≤ 1 year) report problems in various domains, which could reflect previous multi-organ involvement. Examples include decreased exercise intolerance (with or without substantiated standardized physical testing and/or abnormalities on cardiac ultrasound), neurocognitive deficits (e.g., fatigue, headaches, muscle weakness, coordination abnormalities, and worse working memory scores), and psychosocial problems with decreased quality of life (QoL) [9–14]. One report showed that certain morbidities seem to be associated with clinical characteristics upon hospitalization [15]. Furthermore, reports of long-term COVID-19 sequelae and their impact in the adult population lead to speculation whether a similar trajectory could exist in children after SARS-CoV-2 infection [16].

The primary aim of this study was to investigate functional, neurocognitive, psychosocial, and QoL outcomes in MIS-C patients 24 months after PICU admission. The secondary aim was to compare these multidimensional outcomes between 3 and6 months and 24 months after MIS-C PICU admission.

## Materials and methods

### Study design and participants

This is a prospective cohort study of MIS-C patients admitted to the PICU of Erasmus MC Sophia Children’s Hospital, a tertiary care hospital, in Rotterdam, The Netherlands. All MIS-C patients admitted between May 2020 and March 2022 to the PICU were eligible for this study. The diagnosis of MIS-C was made per the World Health Organization (WHO) case definition [3]. The Medical Research Ethics Committee Erasmus MC approved (METC-2021–0538) under the Medical Research Involving Human Subjects Act under Dutch law (known by the Dutch abbreviation WMO). Written consent was obtained from parents, caretakers, and/or patients aged 12 years or older for the use of anonymized data. A subset of patients was included previously in a short-term nationwide follow-up study at 3–6 months after PICU discharge [12].

All patients were invited for follow-up at 3–6 months and 24 months after PICU discharge as part of a standard of care post-PICU follow-up program. At both follow-up visits, patients were seen by a pediatric intensivist (CB) (semi-structured interview and physical exam) and a neuropsychologist (extensive neurocognitive tests and questionnaires completed by one of the parents and/or the child when age-appropriate). Patients were excluded from follow-up when they reached the age of 18 years.

### Demographical and admission characteristics

The following data regarding PICU admission were prospectively collected: [1] demographic characteristics (i.e., sex and age at admission); (2) admission characteristics (i.e., disease characteristics and markers of illness severity such as peak laboratory values, first PICU cardiac ultrasound findings and peak vasoactive-inotropic score); (3) treatment characteristics (i.e., respiratory support and immunomodulation therapies); and (4) PICU outcome (i.e., mortality, PICU length of stay, and functionality scores at PICU discharge).

### Functional outcomes

At 3–6 months and 24 months, follow-up physical examination was performed, and anthropometric data (weight, length) were collected. The following items were evaluated by a pediatric intensivist in a semi-structured interview:

1. Self-reported complaints (i.e., physical, psychosocial, neurocognitive, and/or other)
2. Use of healthcare services and/or psychological care
3. Social participation (i.e., school attendance, hobbies)
4. Global functionality scores

Three global functionality assessments were used: functional status score (FSS), pediatric cerebral performance category (PCPC), and pediatric overall performance category (POPC). Outcome categorization was as follows: FSS was dichotomized into “favorable” outcome (score 6–7) and “unfavorable” outcome (score ≥ 8). PCPC and POPC were dichotomized into “favorable” outcome (score 1 or 2) and “unfavorable” outcome (score 3–6).

### Neurocognitive outcomes

A broad range of neuropsychological domains was tested, using age-appropriate, validated standardized neuropsychological tests at 3–6 months and 24 months follow-up.

Tested domains included the following:

1. General intelligence (Wechsler scales of intelligence) [17–20]
2. Visual-motor integration (Beery Developmental Test of Visual Motor Integration and Rey-Osterrieth Complex Figure) [21]
3. Verbal memory (15 Words Test)
4. Visual memory (Rey-Osterrieth Complex Figure test) [22]
5. Selective attention (Stroop Color Word Test) [23]
6. Sustained attention (Bourdon Vos) [24]
7. Cognitive flexibility (Trail Making Test) [25]
8. Planning and organization (Key Search Test and Six Elements Test of the Behavioral Assessment of the Dysexecutive Syndrome in Children) [25]

A detailed description of the neuropsychological test battery is shown in Supplementary Table 1. Parents completed a questionnaire regarding the executive functions of the child (Behavior Rating Inventory of Executive Function [BRIEF]) [26].

### Psychosocial and quality of life (QoL) outcomes

Patients and parents completed questionnaires regarding patient psychosocial functioning and QoL at 3–6 months and 24-month follow-up. The domains assessed included the following:

1. Posttraumatic stress symptoms (The Children’s Revised Impact of Event Scale) [27]
2. Emotional and behavioral problems (The Strengths and Difficulties Questionnaire) [28, 29]
3. Health-related quality of life (Pediatric Quality of Life Inventory) [30, 31]
4. Sleep (PROMIS Pediatric Short Form v1·0—Sleep-Related Impairment 8a) and fatigue (PROMIS Pediatric Short Form v2·0—Fatigue 10a) [32, 33]

The following psychosocial outcomes were assessed in the parents of MIS-C patients about themselves:

1. Posttraumatic stress symptoms (post-traumatic stress disorder checklist for Diagnostic and Statistical Manual of Mental Disorders, Fifth Edition) [34]
2. Anxiety (PROMIS SF v1·0—Anxiety 8a), and depression (PROMIS SF v1·0—Depression 8b) [35–37]

### Statistical analyses

Continuous variables are presented as mean with standard deviation (SD) or median with interquartile range [IQR]. Categorical variables are presented as number, proportion, or percentage. *P*-values of < 0.05 were considered statistically significant (two-sided test). Whole group analyses were performed with independent *t*-tests for numerical data and chi-squared tests and Fisher’s exact test for categorical data. The differences in neurocognitive test scores and questionnaire scores among patients with MIS-C were compared with normative scores from the general population using either nonparametric or parametric one-sample *t* tests. Repeated measures were analyzed with paired *t*-tests and Wilcoxon signed ranks test for numerical data and McNemar’s tests for categorical data. Analyses were performed in IBM SPSS Statistics version 28.0.1.0 for Windows.

## Results

### Demographic and PICU admission characteristics

**Fig. 1:**
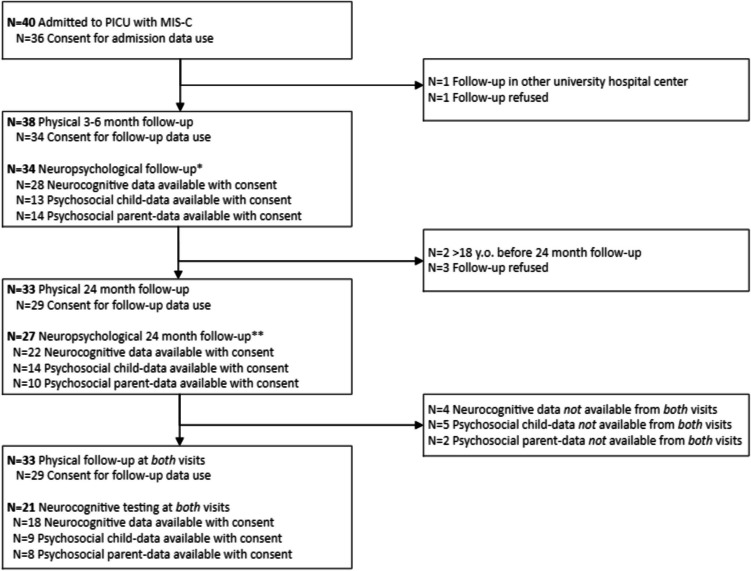
PICU, pediatric intensive care unit; MIS-C, multisystem inflammatory syndrome in children. *No neuropsychological follow-up due to the following reasons: language barrier (*n* = 1); pre-existent psychomotor retardation (*n* = 1); non-testable due to psychological complaints (*n* = 1); neurocognitive testing refused (*n* = 1). **Six patients only participated in physical follow-up, and not in neuropsychological testing due to the following reasons: pre-existent psychomotor retardation (*n* = 1); neurocognitive testing refused (*n* = 5)

Between May 2020 and March 2022, 40 MIS-C patients were admitted to the PICU (Fig. 1). Thirty-six MIS-C PICU admissions consented to use demographic and baseline PICU clinical characteristics (Table 1 and Supplementary Table 2). No significant differences in demographic or clinical characteristics were observed between the cohorts at the evaluation time points, PICU admission, 3–6 months, and 24 months follow-up based on consent rates.

**Table 1:** MIS-C demographic and PICU clinical characteristics

	PICU MIS-C cohort (*N* = 36)		PICU MIS-C cohort with 2-year follow-up (*N* = 29)	
	**Median *[IQR]* or n *(%)***		**Median *[IQR]* or n *(%)***	
	***N***		***N***	
**Sex, male**	36	26 (72)	29	21 (72)
**Age at admission, years**	36	10.1 [7.9–13.6]	29	9.4 [7.7–12.3]
**Prior medical history**	36	11 (31)	29	7 (24)
**Anthropometry**				
Length, cm	35	149 [134–162]	29	141 [134–160]
Weight, kg	36	35.3 [26.5–56.0]	29	34.0 [26.0–49.0]
Overweight (BMI > 1SD)	35	15 (43)	29	11 (38)
Obesity (BMI > 2SD)	35	7 (20)	29	4 (14)
**PICU admission reason**				
(Imminent) Circulatory failure	36	31 (86)	29	24 (83)
Respiratory failure	36	1 (3)	29	1 (3)
Other	36	4 (11)	29	4 (14)
**Cardiac function**				
Shortening fraction, %	35	26.2 [24.1–31.5]	28	26.1 [24.0–30.7]
Shortening fraction, Z-score	29	− 3.0 [− 3.9– − 1.2]	22	− 3.5 [− 4.2– − 1.9]
**Supportive treatment**				
Inotropic/vasopressor support	36	30 (83)	29	24 (83)
Peak Vasoactive-Inotropic Score	36	6.0 [5.0–10.0]	29	5.0 [5.0–10.0]
No respiratory support	36	17 (47)	29	13 (45)
Non-invasive respiratory support	36	19 (53)	29	16 (55)
Invasive respiratory support	36	0 (0)	29	0 (0)
ECMO	36	0 (0)	29	0 (0)
**Anti-inflammatory/anti-coagulation medication**				
IVIG	36	36 (100)	29	29 (100)
Steroids	36	29 (81)	29	24 (83)
Aspirin	36	6 (17)	29	2 (7)
NSAID	36	28 (78)	29	23 (79)
LMWH	36	31 (86)	29	23 (79)
**PICU stay, days**	35	3 [2–4]	29	3 [2–4]
**Post-PICU hospital stay, days**	35	3 [2–4]	29	3 [2–4]
**FSS ‘Favorable’ at PICU-discharge**	35	31 (89)	29	25 (86)
**PCPC “Favorable” at PICU-discharge**	35	32 (91)	29	26 (90)
**POPC “Favorable” at PICU-discharge**	35	20 (57)	29	15 (52)
**Mortality**	36	0 (0)	29	0 (0)

There were no significant differences between included patients at PICU admission and 24-month follow-up. *BMI* body mass index, *ECMO* extra-corporeal membrane oxygenation, *FSS* functional status score, *IVIG* intravenous immunoglobulins, *IQR* interquartile range, *LMWH* low-molecular weight heparin, *MIS-C* multisystem inflammatory syndrome in children, *NSAID* non-steroidal anti-inflammatory drug, *PCPC* pediatric cerebral performance category, *POPC* pediatric overall performance category, *PICU* pediatric intensive care unit, *SD* standard deviation. Categorization functional outcome scores: “Favorable” FSS = 6–7, “Favorable” PCPC = score 1 or 2, “Favorable” POPC = score 1 or 2

### Functional outcomes at 24-month follow-up

Physical examination showed no apparent abnormalities, although 21% (5/24) reported an episode of hair loss. Overweight (8/20, 40%) and obesity (5/20, 25%) persisted in the majority. Thirteen patients (13/29, 45%) reported having at least one chronic physical complaint since PICU discharge. Table 2 provides an overview of self-reported complaints. Our patients consulted physiotherapists (*n* = 6), psychologists/trauma therapists (*n* = 6), occupational therapists (*n* = 2), social workers (*n* = 2), and a dietician (*n* = 1) for complaints that arose after MIS-C hospitalization.

**Table 2:** Longitudinal functional outcomes PICU MIS-C cohort

		3–6 months(*N* = 34)		24 months(*N* = 29)
		*n* (%)		*n* (%)
	*N*		*N*	
**Self-reported**				
Decreased exercise tolerance	34	12 (35)	29	5 (17)
Cardiopulmonary complaints on exertion*	33	4 (12)	27	1 (4)
Chronic fatigue	26	10 (38)	26	6 (23)
Impairments due to chronic headaches	7	5 (71)	5	3 (60)
Increased or decreased appetite	31	7 (23)	24	3 (13)
Smell or taste changes	30	1 (3)	27	0 (0)
Hair loss	27	7 (26)	24	5 (21)
**Functional outcome scores**				
FSS “Favorable”	34	34 (100)	28	28 (100)
PCPC “Favorable”	34	30 (88)	28	27 (96)
POPC “Favorable”	34	30 (88)	28	27 (96)

^*^Reported complaints included: chest pain, palpitations, and syncope on exertion

*FSS* functional status score, *IQR* interquartile range, *PCPC* pediatric cerebral performance category, *POPC* pediatric overall performance category

Categorization functional outcome scores: “Favorable” FSS = 6–7, “Favorable” PCPC = score 1 or 2, “Favorable” POPC = score 1 or 2

Regarding school attendance, 25 patients (25/29, 86%) had resumed school full-time. Two patients attended school part-time due to ongoing fatigue. The reasons that two patients had not resumed school 2 years after hospitalization are as follows: one patient had severe self-reported fatigue and the other patient had a prior history of not attending school. Three patients (3/29, 10%) had an adjusted grade level or repeated a grade. The global functionality scores FSS, PCPC, and POPC were favorable in most patients (100%, 96%, and 96%, respectively).

### Neurocognitive outcomes at 24-month follow-up

Overall, the total mean intelligence score in this cohort was 101 (SD 12) (Table 3). Patients scored within the normal range for most neuropsychological tests: working memory, processing speed, visual-motor integration, verbal memory, selective attention, and executive function. They scored lower than the norm population for short-term visual memory, long-term visual memory, and sustained attention (tempo fluctuations), *p* < 0.05.

**Table 3:** MIS-C neurocognitive outcomes

Outcome variable	*N*, 3–6 months	Mean (SD), 3–6 months	*N***,** 24 months	Mean (SD), 24 months	*N*, Repeated measures	*P*-val**ue**	Deviant Score*, *N*(%), 3–6 months	Deviant Score, *N*(%), 24 months
Intelligence, standard scores with norm mean 100 SD 15*								
Total intelligence quotient	28	96.9 (12.1)	21	101 (11.8)	17	0.337	10%	9%
Working memory index	25	100.1 (11.4)	21	101.8 (12.4)	15	0.889	7%	4%
Processing speed index	24	**95.8 (13.1)**	21	102.2 (13.3)	14	0.083	15%	9%
Visual-motor integration z-scores with norm mean 0 SD (1)*								
Visual-motor integration (VMI Beery)	25	− 0.19 (0.68)	22	− 0.41 (0.88)	18	**0.044**	11%	25%
Visual-motor integration (Rey Complex Figure Copy)	23	0.18 (0.56)	20	− 0.31 (0.95)	14	0.109	4%	20%
Memory, z-scores with norm mean 0 SD (1)*								
Verbal memory, immediate (15 words test)	25	0.02 (1.22)	20	− 0.05 (1.12)	15	0.733	25%	18%
Verbal memory, delayed (15 Words Test)	24	0.09 (1.11)	20	− 0.41 (1.23)	15	0.156	15%	27%
Verbal memory, recognition (15 Words Test)	23	− 0.21 (1.24)	11	− 1.12 (1.97)	8	0.497	13%	36%
Visual memory, immediate (Rey Complex Figure)	21	− **0.70 (1.11)**	20	− **0.75 (1.18)**	13	0.665	33%	50%
Visual memory, delayed (Rey Complex Figure)	22	− **0.68 (1.11)**	20	− **0.79 (1.33)**	13	0.694	28%	50%
Visual memory, recognition (Rey Complex Figure)	22	0.24 (0.94)	19	0.04 (1.34)	13	0.583		
Attention, z-scores with norm mean 0 SD (1)*								
Selective attention (Stroop Color Word Test)	22	0.16 (1.42)	19	0.07 (1.30)	14	0.754	28%	24%
Sustained attention, response time (Bourdon-Vos)	23	− **1.82 (1.95)**	20	− 0.70 (1.27)	14	0.300	62%	32%
Sustained attention, tempo fluctuations (Bourdon-Vos)	23	− **4.62 (4.82)**	20	− **2.00 (2.64)**	14	0.300	77%	50%
Executive functioning z-scores with norm 0 SD (1)*								
Cognitive flexibility (Trail Making Test)	22	− 0.06 (0.91)	19	0.20 (0.89)	14	0.258	12%	10%
Strategy formation (BADS Key Search Test)	22	0.11 (1.16)	18	0.08 (1.03)	12	1.000	17%	15%
Planning skills (BADS Six Elements Test)	16	− **0.70 (0.71)**	12	−0.16 (0.67)	6	**0.042**	31%	7%
**Parent-reported executive functions,** T-score with norm mean 50 SD 10**								
Behavior regulation index	10	47.2 (9.4)	12	48.2 (13.3)	8	0.866	10%	25%
Metacognition index	10	45.6 (9.6)	12	49.2 (11.7)	8	0.944	0%	33%
Total executive function score	11	46.1 (9.6)	12	47.8 (12.9)	8	0.752	9%	17%

*BADS* Behavioral Assessment of the Dysexecutive Syndrome; *MIS-C* Multisystem Inflammatory Syndrome in Children; *SD* Standard Deviation, *VMI* Visual Motor Integration

Bold text: *P*<0.05 difference with standardized norms. *% deviant scores are defined as % of children with MIS-C scoring more than 1 SD lower than the norm score. * A higher score indicates better performance, ** a higher score indicates more problems

### Psychosocial and QoL Outcomes in patients at 24-month follow-up

Regarding psychosocial outcomes in MIS-C patients (*n* = 7), their parents reported significantly (*p* = 0.03) more total emotional and behavioral problems compared with patients in the general population. MIS-C patients’ self-reported QoL scores, as well as fatigue and sleep-related problems, were comparable to the norm (Table 4 and Supplemental Table 3). None of the parents reported increased PTSD risk in their child, and one child (9%) self-reported increased PTSD risk.

**Table 4:** Psychosocial outcomes and quality of life in MIS-C patients and parents

	*N*, 3–6 months	Mean (SD), 3–6 months	*N*, 24 months	Mean (SD), 24 months	Norm data mean (SD)	Repeated measures	*p*-value
**Outcome variable**							
** Posttraumatic stress**							
Total score (parent-reported)	13	21.3 (16.5)	10	11.4 (8.9)^a^	20.2 (14.1)	8	**0.03**
Elevated risk for PTSD (parent-reported), *n* (%)	13	4 (31%)	10	0 (0%)	16%		
Total score (self-reported)	10	26.6 (16.4)	11	**12.5 (12.8)**^**a,#**^	19.5 (13.1)	8	0.07
Elevated risk for PTSD (self-reported), *n* (5)	10	5 (50%)	11	1 (9%)			
** Emotional and behavioral problems in children**							
Total difficulties (parent-reported) z-scores	12	**2.2 (1.0) $**	10	**2.0 (1.1) $**	0 (1)	8	0.14
Total difficulties (self-reported) mean-scores	5	**22.0 (2.1)$**	7	**18.3 (3.1)**^**b**^** $**	8.1 (4.8)		
** Psychosocial outcomes in parents**							
Posttraumatic stress							
Indication for posttraumatic stress disorder, *n* (%)	14	3 (21%)	10	0 (0%)^b^	7%	8*	
Anxiety and depression							
Anxiety symptoms	12	53·0 (11·5)	9	45.4 (9.4)^b^	49.9 (10.1)	6	0.47
Depressive symptoms	12	**46.1 (12.1) $**	9	45.2 (10.0)^b^	49.6 (10.0)	6	0.66
** Health-related quality of life in z-scores (parent-reported)**							
Physical functioning	9	** − **0·46 (1.10)	14	** − **0.94 (1.9)	0 (1)	8	0.48
Emotional functioning	9	** − **0·31 (1.13)	14	0.13 (1.31)	0 (1)	8	0.67
Social functioning,	9	0·03 (0.95)	14	0.08 (0.97)	0 (1)	8	0.53
School functioning	9	** − 0.81 (0.70)**^**c**^	14	** − **0.67 (1.15)	0 (1)	8	0.60
** PROMIS-Short forms**							
Fatigue, T-score	11	45.3 (11.8)	12	37.9 (10.4)	39.8 (12.1)	9	0.16
Sleep-related impairment, T-score	11	51.6 (9.3)	12	44.3 (7.9)	47.5 (10.0)	9	**0.01**

*MIS-C* multisystem inflammatory syndrome in children, *PROMIS* Parent-Reported Outcomes Measurement Information System, *PTSD* post-traumatic stress disorder, *SD* standard deviation

For all outcomes, a higher score is worse (more problems or symptoms)

^a^Norm population is trauma-exposed children and adolescents

^b^Norm population is the Dutch general population

^c^Significantly different from norm population, *p* = 0.016

^*^The 8 parents with repeated measures had no indication for PTSD on both follow-up moments; *p* =.03 compared with norm data. $ = *p* <.01 compared with norm data

### Psychosocial outcomes in parents at 24-month follow-up

Parents (*n* = 10) reported no increased risk for PTSD and comparable depressive and anxiety symptoms (*n* = 9) in themselves compared to the norm (Table 4).

### Outcome comparison between 3, 6, and 24-month follow-up

No significant changes were reported regarding global functional scores (FSS, PCPC, POPC) or physical problems (e.g., fatigue and exercise intolerance) from 3 to 6 months to 24 months (Table 2). As to neurocognitive outcomes, overall, no changes in most neurocognitive domains were found, except for lower scores for visual motor integration (*p* = 0.044) and improved scores on planning skills (*p* = 0.042) over time (Table 3). Overall, psychosocial and QoL outcomes remained stable over time. Parent-reported PTSD symptoms in MIS-C patients decreased over time (*p* = 0.03). This was mainly because of fewer intrusion symptoms (*p* = 0.04). Self-reported scores for sleep-related impairments improved over time (*p* = 0.01) (Table 4).

## Discussion

This is the first long-term longitudinal, prospective study that compared functional and neuropsychological outcomes at 3–6 months and 24 months in a homogenous cohort of PICU MIS-C patients. Their critical illness course was relatively short (median 3 days), and all patients survived. Repeated validated neuropsychological testing showed worse scores on visual memory and sustained attention domains compared to norms. However, in general, there were favorable longitudinal multidimensional outcomes in the majority of our MIS-C PICU cohort. In contrast, self-reported complaints were frequent (45%), and 14% had not resumed full-time school. Parents reported more emotional and behavioral problems.

In general, there is increasing recognition of post-PICU problems in various domains (psychological, physical, and cognitive), which has led to the conceptualization of the post-intensive care syndrome in pediatric (PICS-p) framework. This system helps identify post-PICU sequelae and holds the potential to facilitate patient-specific rehabilitation. Our MIS-C PICU cohort had a relatively good functional outcome and predominantly normal neurocognitive development over time as measured by a validated neurocognitive testing battery. Only visual memory and sustained attention scores remained significantly lower than the norm scores over time. Overall, the neurocognitive outcomes are reassuring given the high incidence of neurological symptoms upon PICU presentation (58%, 12/36). This is in contrast with a recent study by Francoeur et al. that reported 28% of MIS-C survivors had new neurocognitive and/or functional morbidity at hospital discharge [15]. Differences with our study are their case mix of PICU and non-PICU MIS-C admissions and characterization by medical record instead of in-person. Also, FSS, PCPC, and POPC scores are relatively crude scores to determine functional and neurocognitive functioning. Our outcomes may be attributed to the relatively short PICU course without fatalities. This differs from other MIS-C studies, with or without PICU admission, reporting mortality rates between 0.8 and 8% [6–8, 13, 38–40]. The only report of solely PICU MIS-C patients by Davies et al. reported a mortality of 2/76 (3%) [41]. The PICU trajectory described in our study could be the result of a heightened national awareness of this potentially fatal disease. Our nationwide pediatric care network was primed to promptly refer MIS-C patients to a PICU if signs of circulatory instability developed. PICU therapeutic strategies in our cohort were as recommended in the international MIS-C consensus statement [7, 42, 43], with swift initiation of anti-inflammatory medications, if not already administered in the referring hospital. Timely commencement and add-on of anti-inflammatory medications may have influenced disease severity and subsequent PICU and hospital course, as also suggested by the RECOVERY Collaborative Group immunomodulatory therapy study [44]. There are hypotheses that MIS-C and long COVID, both classified as post-acute sequelae of COVID-19 (PASC), share etiological hyper-inflammatory similarities [45]. It is therefore conceivable that the timely initiation of anti-inflammatory agents could positively influence (long-term) outcomes, such as in our cohort. This might be reflected by the fact that our cohort already fared well at the 3–6-month follow-up as measured by validated and detailed neurocognitive evaluation.

However, the prevalence of self-reported complaints, such as chronic fatigue with decreased exercise tolerance and frequent headaches, is high, and health care utilization at 24 months is prevalent. These observations are not uncommon when compared to other MIS-C cohorts [14, 15, 46]. Parents in our study reported high rates of long-term emotional and behavioral problems in their children after PICU discharge. Possible factors contributing to these findings could include heightened parental vigilance and stress during the COVID-19 pandemic and lack of school structure due to lockdowns. It must be noted that interpretation of these parent-reported data should be with caution due to small numbers. Nonetheless, our findings are comparable to outcomes of both community studies during the COVID-19 pandemic and studies in other PICU populations [47, 48]. COVID-19 community studies showed an increased (and persisting) incidence of mental health problems among patients and adolescents [48]. Studies in other PICU populations highlight the increased prevalence and significant impact of anxiety, depression, and posttraumatic stress disorder (PTSD) frequently observed following PICU admission [47]. These could be contributing factors to the worrying fact that a substantial portion of our cohort had not resumed school (14%) or had an adjusted school grade (10%). The worse neurocognitive outcomes in our cohort regarding persistent impaired delayed visual memory and sustained attention scores over time could also be contributing factors. These domains are essential for school performance: paying attention, remembering information, and keeping up with school work. Deficits in these neurocognitive domains have been reported in other MIS-C outcomes studies [48, 49]. Furthermore, we found that a large number of our MIS-C patients were overweight or obese and remained so over time. This suggests potential (pre-existing) lifestyle factors which could compound certain complaints [40, 50]. Such observations could serve as specific targets for individualized rehabilitation efforts of both patients and parents.

Regarding functional outcomes, general PICU outcome studies describe risk factors for impaired long-term functional outcome, which include illness severity, length of PICU stay, and the presence of pre-existing conditions (mostly in the chronic respiratory and/or neurological domains) [51–53]. By comparison, our MIS-C PICU cohort had a relatively short PICU length of stay (median 3 days) and were mostly healthy children. The normalization of functional scores (FSS, PCPC, and POPC) within the first year could reflect the absence of previously described PICU outcome risk factors. In this light, the discrepancy between self-reported and validated testing is of interest and reflects the major difficulty in studies to date: distinguishing disease-related factors from socioeconomic factors in general and specifically the impact of social (restriction) factors during lockdown for MIS-C resulting in social isolation and increased anxiety.

Considering the above, the main limitations of our study are the lack of an appropriate control group and standardized physical testing. However, our study provides unique insights into the growing body of knowledge regarding outcomes after PICU admission for different disease cohorts. The demographic characteristics of our MIS-C cohort are comparable to other MIS-C outcome studies [15, 40, 54], rendering our long-term outcome data of interest when comparing differing MIS-C (PICU) outcome trajectories. This provides insight into the effect of disease-related therapeutic measures on outcome. Our study also underlines the hypothesis that there might be certain baseline patient characteristics (e.g., immunological, anthropometric, and presenting clinical features) that make individuals more prone to developing a critical care trajectory. These observations could facilitate risk assessment and patient stratification, aiding in more optimal individualized therapies, counseling, and rehabilitative efforts. Mostly, our study also emphasizes the importance and feasibility of an ongoing, structured, long-term post-PICU follow-up program. In the Dutch setting, this was facilitated by the presence of a national guideline for post-PICU follow-up programs [55]. The high follow-up rates in our study (> 80%) underline the notion that patients and their families are willing to participate, especially when a novel disease is concerned. As such, our long-term follow-up contributes to a better (insight into the) MIS-C disease trajectory.

In conclusion, despite the initial critical illness, most of our MIS-C PICU admissions did not have significant deficits as scored by validated functional scores and neurocognitive testing. In contrast, self-reported complaints (fatigue and decreased exercise intolerance) remained prevalent, and educational level adjustments were reported. The interpretation of these findings is challenging and influenced by multiple factors, including the MIS-C disease process, the PICU admission itself, socioeconomic factors, and the impact of COVID-19-related societal measures during the follow-up period. An age- and community-matched control group could have provided further insight into the association of our outcomes with these factors and is a study limitation. However, this study demonstrates the merit of a multidisciplinary, structured, and repeated follow-up program of (novel) life-threatening pediatric diseases in general. Insights gained provide valuable information on outcome trajectory, resulting in a better understanding of the overall disease process, potential patient stratification with appropriate patient and parent counselling, and enable implementation of timely rehabilitation programs to ensure ongoing social and school participation.

## Supplementary Information

Below is the link to the electronic supplementary material.

MOESM 1 (DOCX 34 KB)

## Data Availability

No datasets were generated or analysed during the current study.

## Abbreviations

FSS: Functional status scale
MIS-C: Multisystem inflammatory syndrome in children
PCPC: Pediatric cerebral performance category
PICU: Pediatric intensive care unit
POPC: Pediatric outcome performance category
QoL: Quality of life
SARS-CoV-2: Severe acute respiratory syndrome-coronavirus-2
