# Supplementary material for: Longitudinal functional and neuropsychological 2-year follow-up after intensive care admission for multisystem inflammatory syndrome in children
Source: Eur J Pediatr. 2025 Sep 15;184(10):617. doi: 10.1007/s00431-025-06396-y (PMC12436537; doi:10.1007/s00431-025-06396-y)
Supplement: Supplementary file 1 — (DOCX 34 KB) [file 431_2025_6396_MOESM1_ESM.docx]

**Supplemental Tables**

Supplemental Table 1. Neurocognitive test battery overview

|  | **Test** | **Age (yrs.)** | **Test mean and**  **standard deviation (SD)** | **Notes** | **Deviant score cut-off value** |
| --- | --- | --- | --- | --- | --- |
| General intelligence | Wechsler Preschool and Primary Scale of Intelligence (WPPSI-III) | 2.6-6 | Mean 100, SD 15 | Intelligence quotient standard scores. Higher scores represent better functioning. Total IQ score is based on WPPSI-III TIQ, WISC-V TIQ, or WAIS-IV TIQ. The Verbal Comprehension index is based on: WPPSI-III VIQ, WISC-V VCI, WAIS-IV VCI.  Deviant scores are interpreted as ≤ 85 | ≤ 85 |
|  | Wechsler Intelligence Scale for Children (WISC-V) | 7-15 | Mean 100, SD 15 |  |  |
|  | Wechsler Adult Intelligence Scale (WAIS-IV) | 16-18 | Mean 100, SD 15 |  |  |
| Verbal memory:  immediate recall | 15 Words Test | ≥6 | Mean 0, SD1 | Z-score compared with age appropriate scores: Higher scores represent better functioning | ≤ - 1 |
| Verbal memory:  delayed recall | 15 Words Test | ≥6 | Mean 0, SD1 | Z-score compared with age appropriate scores: Higher scores represent better functioning |  |
| Selective attention | Stroop Color Word Test (Stroop) | ≥11 | Mean 0, SD1 | Z-score; Higher scores represent better functioning | ≤ - 1 |
| Sustained attention | Bourdon Vos cancellation test | ≥6 | Mean 0, SD1 | Z-score compared with age appropriate scores; Higher scores represent better functioning | ≤ - 1 |
| Visual-Motor Integration | Beery Developmental Test of Visual Motor Integration (Beery-VMI) | ≥2 | Mean 0, SD1 | Z- score: Higher scores represent better functioning | ≤ - 1 |
| Visual memory:  3 minutes recall | Rey-Osterrieth Complex Figure test (Rey CFT) | ≥6 | Mean 0, SD1 | Z-score: Higher scores represent better functioning | ≤ - 1 |
| Visual memory:  30 minutes recall | Rey-Osterrieth Complex Figure test (Rey CFT) | ≥6 | Mean 0, SD1 | Z-score: Higher scores represent better functioning | ≤ - 1 |
| Executive functions: flexibility | Trail Making Test part B (TMT-B) | ≥8 | Mean 0, SD1 | Z-score compared with age appropriate scores: Higher scores represent better functioning | ≤ - 1 |
| Executive functions: Strategy formation | Behavioral Assessment of the Dysexecutive Syndrome in Children  (BADS-C) key search | ≥8 | Mean 0, SD1 | Z-score: higher scores represents better functioning. | ≤ - 1 |
| Executive functions: Planning | Behavioral Assessment of the Dysexecutive Syndrome in children  (BADS-C) six parts test | ≥8 | Mean 0, SD1 | Z-score: higher scores represents better functioning. | ≤ - 1 |

Supplemental table 2. PICU MIS-C cohort presenting symptoms and diagnostics

|  | PICU MIS-C cohort  (N=36) | | PICU MIS-C cohort 2-year follow-up  (N=29) | |
| --- | --- | --- | --- | --- |
|  |  | **Median [IQR] or n (%)** |  | **Median [IQR] or n (%)** |
|  | **N** |  | **N** |  |
| **Symptoms** |  |  |  |  |
| Fever | 36 | 36 (100) | 29 | 29 (100) |
| (Muco)cutaneous Signs ^a^ | 36 | 29 (81) | 29 | 24 (83) |
| Respiratory Distress | 36 | 13 (36) | 29 | 10 (35) |
| Shock or Hypotension | 36 | 35 (97) | 29 | 29 (100) |
| Gastro-intestinal symptoms ^b^ | 36 | 36 (100) | 29 | 29 (100) |
| Neurological symptoms^c^ | 36 | 21 (58) | 29 | 19 (66) |
| **Laboratory Values** |  |  |  |  |
| CRP (mg/L) | 35 | 204 [147 – 295] | 28 | 193 [152 – 300] |
| ESR (mm/hr) | 21 | 41 [30 – 91] | 17 | 67 [30 – 91] |
| Ferritin (ug/L) | 35 | 851 [530 - 1495] | 28 | 806 [498 – 1427] |
| IL-6 (pg/ml) | 34 | 145 [62 – 471] | 27 | 144 [63 – 456] |
| PCT (ng/L) | 28 | 15.15 [2.93 – 28.78] | 21 | 19.84 [3.79 – 30.40] |
| D-dimers (mg/L) | 32 | 3565 [2188 – 6375] | 25 | 3820 [2105 – 5880] |
| PT (sec) | 33 | 14.2 [13.1 – 15.7] | 27 | 13.5 [12.0 – 14.9] |
| PT-INR | 32 | 1.3 [1.1 – 1.4] | 26 | 1.2 [1.1 – 1.3] |
| NT-pro-BNP (pmol/L) | 35 | 12144 [7654 – 24694] | 28 | 12478 [7916 - 24034] |
| hsTnT (ng/L) | 35 | 0.080 [0.024 – 0.164] | 28 | 0.081 [0.026 – 0.187] |
| **Microbiology and Serology** |  |  |  |  |
| SARS-CoV-2 RAT, positive | 31 | 3 (10) | 27 | 3 (11) |
| SARS-CoV-2 IgM, positive | 16 | 10 (63) | 13 | 8 (62) |
| SARS-CoV-2 IgG, positive | 12 | 11 (91) | 11 | 10 (91) |
| SARS-CoV-2 Ig total, positive | 16 | 16 (100) | 12 | 12 (100) |
| Any positive serology/RAT present | 36 | 29 (81) | 36 | 23 (79) |
| Blood Cultures, positive ^d^ | 32 | 1 (3) | 30 | 1 (3) |

There were no significant differences between included patients at PICU admission and 24 month follow-up. CRP = C-Reactive Protein, ESR = Erythrocyte Sedimentation Rate, hsTnT = high sensitivity Troponin-T, IgG = Immunoglobulin G, IgM = Immunoglobulin M, IL-6 = Interleukin 6, IQR = interquartile range, MIS-C = multisystem inflammatory syndrome in children, NT-pro-BNP = N-Terminal prohormone of Brain Natriuretic Peptide, PCT = Procalcitonin, PICU = Pediatric Intensive Care Unit, PT = Prothrombin Time, PT- INR = Prothrombin Time, International Normalized Ratio, SARS-CoV-2 = Severe Acute Respiratory Syndrome Coronavirus 2, RAT = Rapid Antigen Test^a^ Non-purulent conjunctivitis, oral changes or skin rash; ^b^ Nausea, vomiting, abdominal pain or diarrhea; ^c^ Lowered GCS, encephalopathy, confusion, convulsions, headaches or neurological deficits; *^d^* Positive test due to contamination.

Supplement Table 3. Psychosocial subscale outcomes, parent-reported

|  | **3-6 months** | | **24 months** | | **Repeated measures** | |
| --- | --- | --- | --- | --- | --- | --- |
|  | **N** | **Mean [IQR]** | **N** | **Mean [IQR]** | **N** | **p-value** |
| **Outcome variable** |  |  |  |  |  |  |
| ***Posttraumatic stress**** |  |  |  |  |  |  |
| Intrusion | 13 | 6·0 [1·0 - 11·5] | 10 | 1·5 [0 – 4·8] | 8 | **0**·**04** |
| Avoidance | 13 | 3·0 [0 – 9·5] | 10 | 0·5 [0 – 4·3] | 8 | 0·75 |
| Hyper-arousal | 13 | 11·0 [4·5 - 13·0] | 10 | 6·0 [3·0 - 12·3] | 8 | 0·15 |
| ***Emotional and behavioral problems**** |  |  |  |  |  |  |
| Emotional symptoms | 12 | 3·5 [0·3 - 7·0] | 10 | 2·0 [1·0 - 4·5] | 8 | 0·38 |
| Conduct problems | 12 | 1·5 [0 – 3·8] | 10 | 0·5 [0 – 2·0] | 8 | 0·40 |
| Hyperactivity/inattention | 12 | 4·0 [3·0 - 6·8] | 10 | 4·5 [2·8 - 7·0] | 8 | 1·00 |
| Peer problems | 12 | 3·0 [2·0 - 4·0] | 10 | 2.5 [2·0 - 3·0] | 8 | 0·80 |
| Prosocial behavior** | 12 | 8·5 [7·3 - 9·8] | 10 | 8·0 [7·8 - 9·3] | 8 | 0·86 |

MIS-C = multisystem inflammatory syndrome in children, IQR = interquartile range

* Higher score = worse (more problems or symptoms); ** higher score = better (more pro-social behavior); 1. norm population is trauma-exposed children and adolescents; 2. z-scores based on specific sex/age groups from the Dutch general population; 3. Norm population is the Dutch general population.
